# Composites Composed of Hydrophilic and Hydrophobic Polymers, and Hydroxyapatite Nanoparticles: Synthesis, Characterization, and Study of Their Biocompatible Properties

**DOI:** 10.3390/jfb12040055

**Published:** 2021-10-01

**Authors:** Mariia Gordienko, Elena Karakatenko, Natalia Menshutina, Marina Koroleva, Ilmira Gilmutdinova, Petr Eremin

**Affiliations:** 1 Chemical and Pharmaceutical Engineering Department, D. Mendeleev University of Chemical-Technology of Russia, 125480 Moscow, Russia; chemcom@muctr.ru; 2 Nanomaterials and Nanotechnology Department, D. Mendeleev University of Chemical-Technology of Russia, 125480 Moscow, Russia; eyrfad@gmail.com (E.K.); mkoroleva@muctr.ru (M.K.); 3 National Research Medical Centre of Rehabilitation and Balneology, Ministry of Health of the Russian Federation, 121099 Moscow, Russia; gilm.ilmira@mail.ru (I.G.); ereminps@gmail.com (P.E.)

**Keywords:** hydrophilic and hydrophobic polymers composites, hydroxyapatite nanoparticles, cell adhesion, cytotoxicity, acute toxicity

## Abstract

The creation of artificial biocomposites consisting of biocompatible materials in combination with bioactive molecules is one of the main tasks of tissue engineering. The development of new materials, which are biocompatible, functional, and also biodegradable in vivo, is a specific problem. Two types of products can be formed from these materials in the processes of biodegradation. The first types of substances are natural for a living organism and are included in the metabolism of cells, for example, sugars, lactic, glycolic, and β-hydroxybutyric acids. Substances that are not metabolized by cells represent the other type. In the latter case, such products should not be toxic, and their concentration when entering the bloodstream should not exceed the established maximum permissible level. The composite materials based on a mixture of biodegradable synthetic and natural polymers with the addition of hydroxyapatite nanoparticles, which acts as a stabilizer of the dispersed system during production of the composite, and which is a biologically active component of the resulting matrix, were obtained and studied. The indirect effect of the shape, size, and surface charge of hydroxyapatite nanoparticles on the structure and porosity of the formed matrix was shown. An in vivo study showed the absence of acute toxicity of the developed composites.

## 1. Introduction

Three-dimensional highly porous composites (scaffolds and matrices) are used to create a local bioactive environment in the implantation of mammalian cells to restore damaged tissue and deliver drugs and proteins, as substrates for growing cells [1,2].

A lot of preparation methods for porous matrices are known, including injection molding [3,4], electrospinning [5,6,7,8], leaching using porogen [9], polymerization of reverse highly concentrated emulsions [10], 3-D printing [11,12], and freeze-drying [13,14,15,16].

Both the bulk and surface properties must be considered during the design of tissue engineering materials. It is important to take into account the degree of hydrophilicity of the matrix surface [9], macro- and microporosity [17,18,19], surface roughness [20], mechanical properties (hardness and elasticity), biodegradation rate, absence of cytotoxicity, and biocompatibility of the matrices with body tissues.

Ceramics [21,22] and polymers (synthetic or natural) [23,24,25,26] matrices are known. Having excellent hardness and porosity, the inorganic materials have a low modulus of elasticity, whereas some polymeric materials have a low hydrophilicity, which reduces adhesion, migration, proliferation, and differentiation of the cells [27]. It seems promising to create composite materials that combine the virtues of inorganics and polymers. In tissue engineering, synthetic (copolymer polylactic and polyglycolic acids (PLGA), polycaprolactone (PCL), poly(methyl methacrylate) (PMMA), polyethylene glycol (PEG), etc.) and natural (gelatin, alginate, collagen, chitosan, etc.) polymers and their compositions are used to produce the materials for tissue engineering [7,27,28,29,30,31,32,33,34,35]. In this work, PLGA, PCL, and sodium alginate were used.

PLGA is an excellent material for tissue engineering. It forms natural metabolites during biodestruction and has acceptable mechanical properties [6,36]. Polycaprolactone is a biodegradable hydrophobic aliphatic polyester with high strength and low destruction rate [37,38]. Alginate is a natural polymer, which has a strength similar to fibroblasts [39]. Alginate has a higher destruction rate compared to synthetic polymers but can increase the hydrophilicity of the material. Their combination allows the creation of materials with the required rate of biodegradation, mechanical strength, and desired hydrophilic surface [6,38]. Despite numerous studies, there are conflicting data on cell adhesion and growth on alginate matrices [40,41]. The majority of authors agree that the absence of active growth centers reduces the ability of cells to adhere and proliferate [39,42,43]. In the present work, hydroxyapatite was used as a bioactive filler, improving the adhesion of cells to matrices.

The amount of hydroxyapatite (HAP) in the composites affects physical and mechanical properties: an increase in the HAP value improves mechanical strength but leads to lower swelling rate, biodegradation rate, and drug release [3,44]. HAP enhances the osteogenic potential of cells due to the osteoinductive response. HAP can neutralize acidic products produced because of biodestruction, preventing the death of cells and tissues due to low pH [45]. There is not enough data on the effect of the size and shape of hydroxyapatite nanoparticles on the mechanical properties of polymer matrices. It is known that the size and shape of nanoparticles play a significant role in the process of cell growth and proliferation [46]. In this work, we investigated the influence of the size, shape, and number of HAP nanoparticles on the mechanical and bioactive properties of composites composed of hydrophilic and hydrophobic polymers and HAP nanoparticles.

## 2. Materials and Methods

### 2.1. Materials

The following reagents were used for the HAP nanoparticles synthesis: Ca(NO_3_)_2_⋅4H_2_O (99%, Merck, Darmstadt, Germany, Cat. No. C2786), Na_2_HPO_4_ (99%, Sigma-Aldrich), NaOH (analytical grade, Himmed, Moscow, Russian Federation), and sodium citrate (analytical grade, Himmed). For the synthesis of polymer matrices, sodium alginate (chemically pure, Ruschim, Moscow, Russian Federation), a 50:50 copolymer of polylactic and polyglycolic acids (99%, Merck), polycaprolactone (99%, Merck), and dichloromethane (analytical grade, Ruschim) were used. All chemicals were of analytical grade and used directly without further purification. HDF human skin fibroblast cell culture (Cell Applications, San Diego, CA, USA, Cat. No. 106K-05a), DMEM culture medium (4500 mg/L) (StemCell Technology, Vancouver, BC, Canada), FBS fetal serum (Gibco, Grand Island, NY, USA), and L-glutamine (StemCell Technology) were used for cytotoxicity and cell adhesion studies.

### 2.2. Synthesis of HAP Nanoparticles

**Figure 1:**
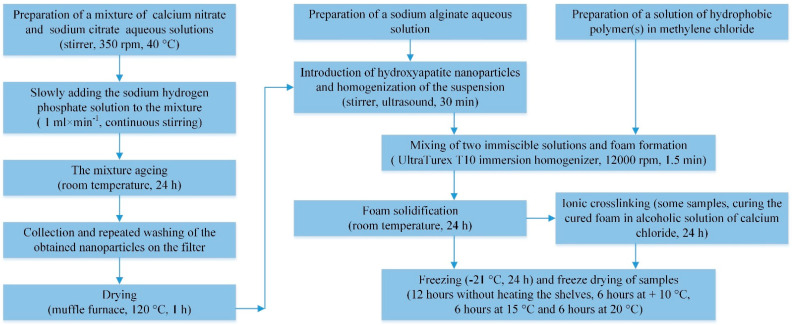
Synthesis of HAP nanoparticles and porous hydrophobic polymer(s)/alginate/HAP NPs matrices.

Hydroxyapatite nanoparticles (HAP NPs) were synthesized by the controlled precipitation method using sodium citrate as a stabilizer according to the Reaction (1) (Figure 1).

(1) $$10\cdot \mathrm{Ca}{\left({\mathrm{NO}}_{3}\right)}_{2}+6\cdot {\mathrm{Na}}_{2}{\mathrm{HPO}}_{4}+8\cdot \mathrm{NaOH}={\mathrm{Ca}}_{10}{\left({\mathrm{PO}}_{4}\right)}_{6}{\left(\mathrm{OH}\right)}_{2}+20\cdot {\mathrm{NaNO}}_{3}+6\cdot H_{2}O$$

A volume of 20 mL of calcium nitrate aqueous solution (0.1 M) was added to 20 mL of a sodium citrate solution (0.15 M). The mixture was stirred in a flask on a magnetic stirrer at 350 rpm and 40 °C. Next, sodium hydrogen phosphate solution (0.06 M) was added to the obtained mixture using a peristaltic pump at a rate of 1 mL⋅min^−1^. The molar Ca/P ratio was 1.67. The pH was kept at 9.0 using sodium hydroxide solution. After adding the required amount of sodium hydrogen phosphate, the mixture was stirred for 30 min and left to age at room temperature for 24 h. The obtained precipitate was filtered, washed three times with double-distilled water, and dried using a muffle furnace at 120 °C for 1 h.

### 2.3. Synthesis of Porous Hydrophobic Polymer(s)/Alginate/HAP NPs Matrices

Porous hydrophobic polymer(s)/alginate/HAP NPs matrices were prepared in 3 steps (Figure 1).

In the first step, sodium alginate powder was dissolved in 9 mL of water so its concentration was 2 wt.%. The mixture was left for 15 min at 50 °C under stirring until the polymer particles swelled, and the obtained medium-viscosity hydrogel became homogeneous. A hydroxyapatite powder weighing 0.05 g, 0.1 g, or 0.15 g was added to the sodium alginate hydrogel and mixed thoroughly. The resulting mixture was subjected to ultrasound for 30 min to uniformly disaggregate and distribute hydroxyapatite nanoparticles in the volume of the alginate hydrogel.

In the second step, a 3 wt.% solution of a biocompatible hydrophobic polymer in methylene chloride with a volume of 3 mL was prepared. Poly(lactic-co-glycolic acid) (PLGA), polycaprolactone (PCL), or their mixtures of PLGA/PCL, taken in a 1:1 ratio, were used as hydrophilic polymers.

In the third step, a dispersed “liquid–gas” system was prepared in which the liquid was a mixture of two immiscible solutions. For this, foaming of the sodium alginate medium-viscosity hydrogel was performed using an UltraTurex T10 immersion homogenizer (IKA, Staufen, Germany) and, at the same time, a hydrophobic polymer solution was poured in a thin stream. Homogenization was carried out at 12,000 rpm for 1.5 min. In this case, hydroxyapatite nanoparticles worked as a stabilizer for the obtained complex dispersed system. The resulting dispersed system was poured into glass molds and left for 24 h at room temperature to evaporate dichloromethane and cure the foamed sample. Additionally, a series of experiments were carried out in which, at this stage, the hardened matrix was kept in an alcoholic solution of calcium chloride to crosslink the polymer chains of alginate. The samples were frozen at −21 °C for 24 h and were freeze-dried to remove solvents under the following temperature conditions ultimately: 12 h without heating the shelves, 6 h at + 10 °C, 6 h at 15 °C, and 6 h at 20 °C.

Brief descriptions of the obtained samples are presented in Table 1.

### 2.4. Measurement of the ζ-Potential of Hydroxyapatite Nanoparticles

The ζ-potential was measured using a Zetasizer Nano ZS laser particle analyzer (Malvern, Worcestershire, UK). The measurements were carried out five times at 25 °C, and then the average result was used for further analysis.

### 2.5. Scanning Electron Microscopy

The survey was carried out using a TESCAN Vega 3 LMU scanning electron microscope (TESCAN, Brno–Kohoutovice, Czech Republic) with a tungsten cathode. The HAP NPs dispersion was treated with ultrasound with a power of 65 W for 30 min for destruction of the possibly formed agglomerates. A drop of dispersion was applied to an electrically conductive graphite tape with an automatic pipette with disposable tips, and it was then dried in vacuum without heating. The survey was carried out at a 10 kV accelerating voltage and 4 mm working distance.

The composites were studied using a TESCAN Vega 3 LMU scanning electron microscope with a tungsten cathode. A thin layer of the sample was placed on a graphite substrate. The survey was carried out at an accelerating voltage of 5 kV, and a working distance of 3.1 mm.

The size distribution of HAP NPs was determined based on statistical data.

For each concentration of surfactants used in the synthesis, the size of at least 100 formed particles was estimated. Histograms of the particle size quantitative distribution were constructed based on the data obtained. In the case of lamellar morphology particles, the length and thickness of the particles were evaluated; in the case of rod-shaped particles, the length and diameter were evaluated.

### 2.6. Fourier-Transform Infrared (FT-IR) Spectra

The spectra of the hydroxyapatite powder in the KBr tablet were recorded on a Nicolet 380 infrared Fourier spectrometer equipped with a Smart Performer single-impaired total-internal-reflection attachment at the Center of Collective Use of MUCTR. The spectrum was recorded from 4000 to 400 cm^−1^. The spectral resolution was 4 cm^−1^ and the total recording time of each spectrum was 1 min. The spectrometer was controlled using the OMNIC 7.3 program (version 7.3.5, Nicolet, Madison, WI, USA).

### 2.7. X-ray Phase Analysis

The phase composition of the samples was determined using a Bruker D2 PHASER X-ray diffractometer (Billerica, MA, USA) using Ni-filtered Cu Kα1 radiation with a graphite monochromator (l = 1.54178 Å). Tube mode (Cu) was used at 10 mA and 30 kV. The X-ray diffraction patterns were recorded in the angular range (2θ) from 20° to 60° with a step size of 0.025°, a slit of 0.6 mm, a shutter speed at the point of 0.5 s (several passes with the data accumulation), and an energy discriminator of 0.17–0.23 keV. The decryption of the spectrum was carried out using the JCPDS-ICDD library with special software (Topas X-ray analysis package, version 6, Bruker, Billerica, MA, USA). For the study of nanoparticles, powders were taken, dried at 120 °C, or calcined at 400 °C. X-ray diffraction patterns of the composites were obtained from a sample rolled into a thin sheet.

### 2.8. Energy-Dispersive Microanalysis

Elemental analysis was performed using an Oxford Instruments X-Max 50 detector (Abingdon, Oxfordshire, UK) with a TESCAN Vega 3 LMU scanning electron microscope. Points for probe analysis were chosen arbitrarily. The value of the accelerating voltage of the electronic probe was 20 kV. A preliminary image was obtained using a BSE phase-contrast detector.

### 2.9. Assessment of the Mechanical Stability of Hydrophobic Polymer(s)/Alginate/HAP NPs Matrices

To assess the mechanical stability of the matrices, the samples were placed in 35 mm Petri dishes, and 2 mL of phosphate-buffered saline was added and left in an incubator at 37 °C in 5% CO_2_. Visual assessment was carried out 72, 96, and 120 h after the start of the experiment using a Zeiss Axio Observer A.1 microscope (Zeiss, Oberkochen, Germany) with an AxioCam MRC5 digital camera (Zeiss, Oberkochen, Germany) using Axiovision software (version 4.8.2, Zeiss, Oberkochen, Germany).

### 2.10. Assessment of Cytotoxicity and Cell Adhesion of Hydrophobic Polymer(s)/Alginate/HAP NPs Matrices

Cytotoxicity studies of hydrophobic polymer(s)/alginate/HAP NPs matrices were performed on a commercial HDF human skin fibroblast cell culture (Cell Applications, San Diego, CA, USA, Cat. No. 106K-05a). Cells were cultured in a CO_2_ incubator (37 °C, 5% CO_2_) in a high-glucose DMEM culture medium (4500 mg⋅L^−1^) supplemented with 10% FBS fetal serum and 2 mmol⋅L^−1^ of L-glutamine. The cell culture was passaged on Petri dishes of d = 35 mm with a density of 104 cells⋅cm^−2^.

The samples were sterilized by UV irradiation, washed 3 times with phosphate-buffered saline, and poured into the culture medium. Samples were left for 8 h at +4 °C. The test samples were placed in Petri dishes with a monolayer culture and left for 72 h in an incubator at 37 °C in 5% CO_2_. After a time, the supernatant was taken and transferred to a new Petri dish for visual assessment of dead cells. Cells in the studied Petri dishes were removed from the surface of the plates and counted with the assessment of viability on an automated counter Countess (Invitrogen, Korea) according to the manufacturer’s method.

To assess the cell adhesion, after 120 h, samples were transferred to a new sterile Petri dish with culture medium and left in the incubator for 7 days at 37 °C in 5% CO_2_. The presence and number of cells near the matrices were estimated visually, and conclusions about the cells’ adhesive property of the hydrophobic polymer(s)/alginate/HAP NPs matrices were made.

### 2.11. Assessment of Acute Toxicity of Hydrophobic Polymer(s)/Alginate/HAP NPs Matrices

The study was performed on sexually mature outbred white mice and Wistar rats. Prior to the study and in its process, the principles of the humane treatment of experimental animals were respected. The animals were kept under controlled environmental conditions at 18–22 °C and 30–70% relative humidity. In addition, a 12 h day–night lighting cycle and a 15-fold change in the air volume of the room per hour were observed in the animal welfare rooms. Standard extruded fodder of the LLC “Laboratorkorm” (Russia) and drinking water were used without restriction for feeding animals.

Extracts of samples were prepared in a model medium. An isotonic solution of sodium chloride 0.9% was used as a model medium. Each test sample was measured before extraction, and its surface area was determined and placed in a 50 mL glass flask, crushed into pieces with an area of not more than 1 cm^2^. The model medium was poured into the flask to the sample at a rate of 1 mL per 3 cm^2^ of the surface area calculated in total from both sides of the sample. The flask with the sample was placed in a dry-air thermostat at 37 °C for 72 h, stirred once daily by shaking the flasks. The indicated extraction temperature was chosen due to the fact that when using the samples for their intended purpose, they are expected to come into contact with cell cultures or body tissues of warm-blooded animals whose temperature is close to 37 °C.

After the flasks with extracts of the test samples were removed from the thermostat, they were cooled to room temperature, shaken vigorously for 30 s, and the contents were poured into dry sterile 10 mL conical tubes. Tubes with extracts were centrifuged at 2000 rpm for 10 min, after which the supernatant was poured into sterile 10 mL glass vials, closed with rubber stoppers, and crimped with aluminum caps. Extract samples were stored in a refrigerator at 2–4 °C prior to injection.

The acute toxicity of sample extracts was studied by intraperitoneal injection to mice and rats. The extract of each sample was injected into five male and five female mice intraperitoneally at a dose of 40 mL per kg body weight (1 mL per animal weighing 25 g) and five male rats intraperitoneally in a volume of 25 mL per kg (5 mL per animal weighing 200 g). A single control was chosen: groups of mice and rats, equal in the number of animals to the experimental groups, which were injected with an isotonic (physiological) solution of sodium chloride 0.9% in the same volumes as the extracts of the samples from experimental mice and rats.

The period of observation of animals was 14 days, during which their clinical condition was evaluated, and body weight was determined (initial, on the 8th and 14th day of the experiment).

Due to the lack of death of animals during the observation period, a necropsy was performed as planned on the 15th day after the introduction of extracts of samples to animals. All animals of the experimental and control groups were subjected to post-mortem autopsy. The organs of the thoracic and abdominal cavities were evaluated visually macroscopically, additionally paying attention to possible morphological signs of a local irritant effect.

When analyzing the results of the study, for the statistical assessment of quantitative data (bodyweight), descriptive statistics were used in the study: the average value and standard deviation were calculated, which are presented in the final tables. Intergroup differences were determined at a confidence level of 95% according to the Mann–Whitney method.

## 3. Results

### 3.1. Characterization of Hydroxyapatite Nanoparticles

**Figure 2:**
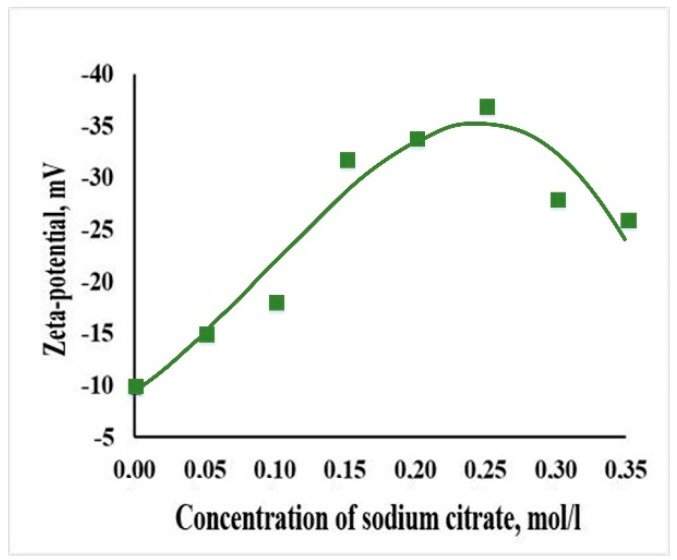
The dependence of the ζ-potential of the HAP nanoparticles dispersion (1 wt.%) in a sodium citrate aqueous solution from concentration at pH = 9.

Sodium citrate plays a significant role in the formation of HAP crystals in the human body. During the synthesis, citrate ions can act as a chelating agent for calcium ions, thereby slowing down the process of crystal formation while stabilizing nanoparticles due to electrostatic repulsion. The dependence of the ζ-potential of the HAP NPs dispersion in a sodium citrate aqueous solution on the concentration of the stabilizer was measured in order to assess the stability of the dispersed system, as well as to determine the magnitude of the charge (Figure 2).

The system had a negative charge at pH = 9. The value of the electrokinetic potential was equal to 10 mV in the absence of a stabilizer. The particle charge sharply increased up to −35 mV while citrate concentration increased up to 0.15 M and reached a maximum of −38 mV at a concentration of 0.25 M (ratio Cit^3−^/Ca^2+^ = 2.5), after which it monotonically decreased to −25 mV. Presumably, the adsorbed citrate ions on the surface of the HAP NPs occurred after the formation of a monolayer. Dispersions of synthesized particles at concentrations from 0.15 M to 0.30 M were aggregative and sedimentation-stable for at least 15 days.

**Figure 3:**
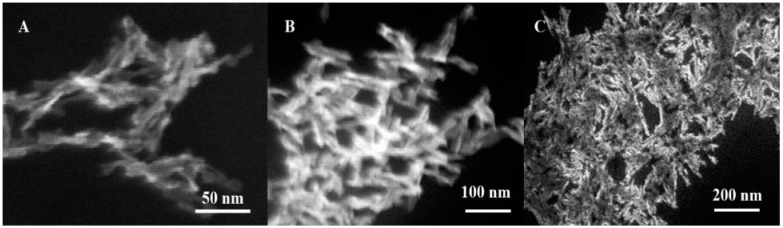
SEM microphotographs of hydroxyapatite nanoparticles obtained in the presence of sodium citrate at concentrations of 0.05 M (**A**), 0.10 M (**B**), and 0.15 M (**C**).

The specific adsorption of citrate by HAP crystals seemed to occur due to the morphology of the formed nanoparticles. Figure 3 shows SEM microphotographs of hydroxyapatite nanoparticles.

The Ca/P molar ratio in all samples of hydroxyapatite nanoparticles was close to stoichiometric (1.67 ± 0.02). This was previously confirmed by a complex of physicochemical methods (X-ray phase analysis, energy-dispersive microanalysis).

At a concentration of sodium citrate equal to 0.05 M, plate-shaped NPs were formed, which upon drying formed agglomerates several micrometers in size. The plates had an average thickness of 4 nm, and a length of 30 to 50 nm. An increase in the stabilizer concentration to 0.10 M led to the formation of rod-shaped nanoparticles with an average diameter of 10 nm and a length of 150–220 nm, which aggregated. In this case, aggregates of less than 1 µm were formed. The morphology of the nanoparticles remained unchanged, and the average length of the nanorods decreased to 120 nm at a concentration of 0.15 M. It should be noted that there was practically no aggregation of nanoparticles.

**Figure 4:**
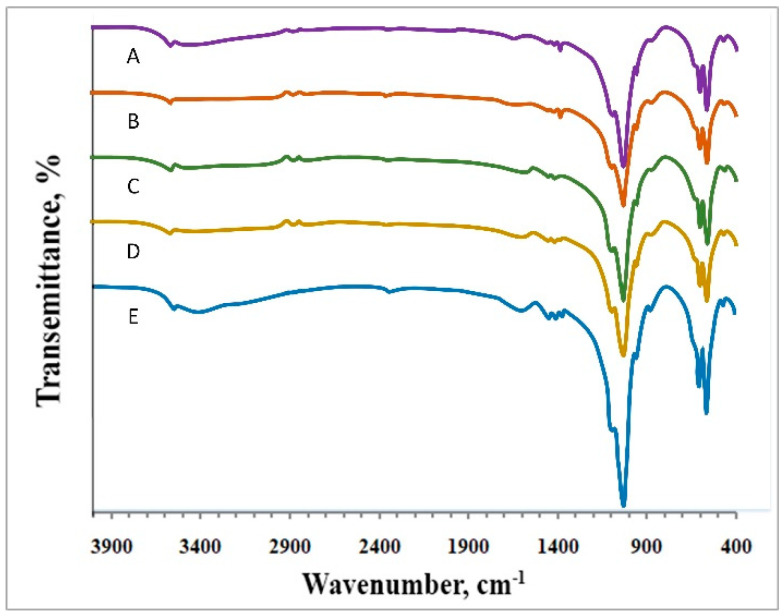
FTIR spectra of HAP NPs obtained at different concentrations of sodium citrate: 0.05 M (**A**), 0.10 M (**B**), 0.15 M (**C**), 0.20 M (**D**), and 0.30 M (**E**).

The FT-IR spectra of HAP NPs are presented in Figure 4.

The FT-IR spectra (Figure 4) show a clear peak at 3572 cm^−1^ and a broadband between 3600 and 1700 cm^−1^, which is attributed to the apatitic OH- group (O-H stretching) and water absorbed during synthesis, respectively [47]. The low peak intensity indicates a small amount of water in the sample. The more pronounced peak appearing at 631.77 cm^−1^ belongs to the apatitic OH group [48]. The broadbands with a peak at 1034 cm^−1^ are attributed to the υ3 bending mode (PO_4_^3−^). The low intense peak at 946 cm^−1^ can be attributed to the υ1 mode of PO_4_^3−^. The bands at 603 and 566 cm^−1^ can be attached to the υ4 bending mode of PO_4_^3−^ [49]. The FT-IR spectra confirmed the formation of an apatite phase with a chemical composition close to that of stoichiometric HAP, confirmed by the XRD data.

A different picture (Figure 4D,E) developed for samples synthesized at higher stabilizer concentrations (0.20–0.30 M). The peaks at 2002 cm^−1^ and 1274 cm^−1^ correspond to the СО_3_^2−^ group. Thus, we can suppose that the studied samples obtained in the presence of sodium citrate with a concentration from 0.20 to 0.30 M were nonstoichiometric carbonate-hydroxyapatite [50]. This phenomenon can be explained by the chelation of calcium ions by citrate anions at elevated concentrations of stabilizer, and the presence of CO_2_ in air and water, which may lead to the substitution of the OH^−^ and PO_4_^3−^ groups by the carbonate anions.

### 3.2. Characterization of Hydrophobic Polymer(s)/Alginate/HAP NPs Matrices

**Figure 5:**
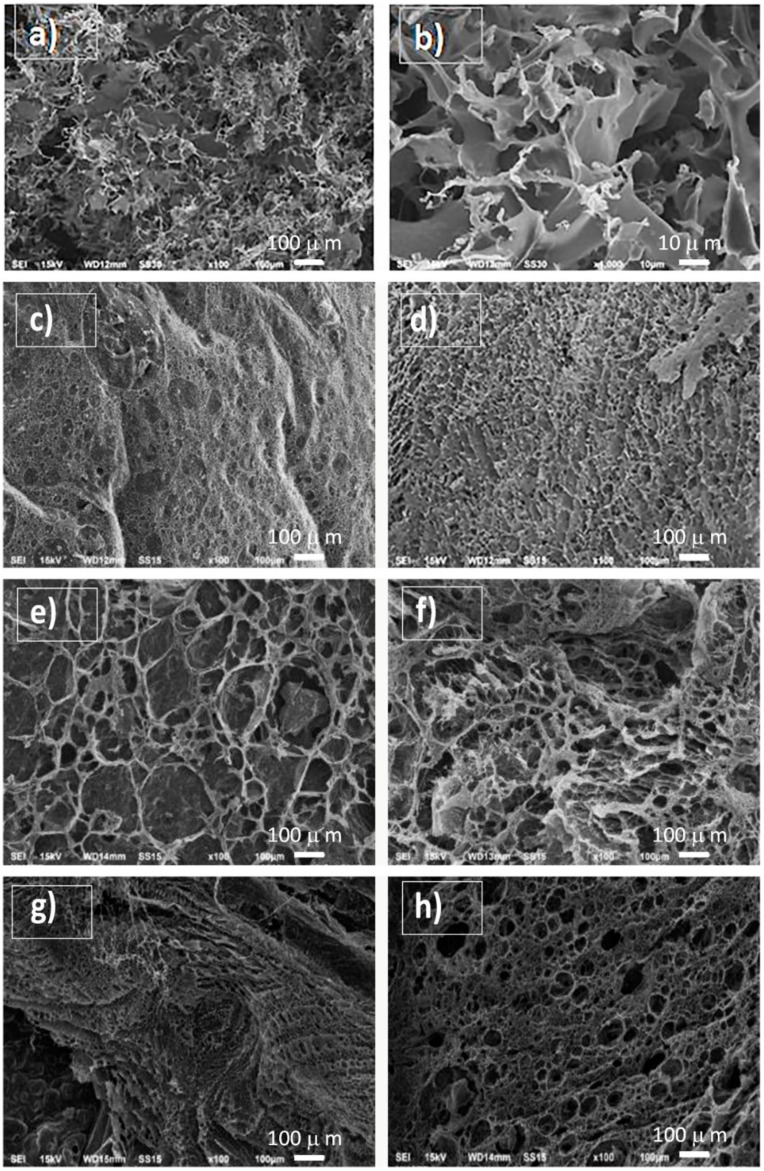
SEM microphotographs of polymer matrixes: without the addition of hydroxyapatite nanoparticles control (**a**,**b**); I-PLGA-0.05 (**c**); I-PLGA-0.10 (**d**); II-PLGA-0.05 (**e**); II-PLGA-0.10 (**f**); III-PLGA-0.05 (**g**); III-PLGA-0.10 (**h**) (see Table 1).

In the studies, three types of HAP NPs were used as a bioactive filler of composite materials: agglomerates consisting of nanoplates (I-PLGA), aggregates consisting of nanorods (II-PLGA), and rod-shaped nanoparticles (III-PLGA). In order to determine the optimal filler concentration, a series of experiments was carried out with the addition of 0.05 or 0.10 g of each type of HAP NPs powder. To compare the results obtained, a control experiment was carried out without adding HAP. PLGA was chosen as a polymer in this series of experiments. Figure 5 shows SEM microphotographs of PLGA/alginate and PLGA/alginate/HAP NPs matrices.

The internal structure of the composite material sample without the addition of HAP NPs (Figure 5a,b) was porous; there were areas with a plateau. Pore sizes ranged from 30 to 80 microns. The wall thickness of the matrix ranged from 2 to 30 microns. No pores were detected in the walls.

I-PLGA series samples (Figure 5c,d) had either low porosity or mainly closed pores. An increase in the content of HAP NPs led to the formation of more stable structures; however, in this case, the filler was distributed unevenly, and hydroxyapatite was present in the sample in the form of agglomerates.

II-PLGA series samples (Figure 5e,f) had a porous three-dimensional structure. Large pores 50–250 μm in diameter formed in the sample (sample II-PLGA-0.05) at a lower content of HAP. Sample II-PLGA-0.10 formed oval pores of a smaller size (30–80 μm). In both cases, the pore walls also had a porous structure. It should be noted that in these samples, the matrix walls in most cases were solid, and there were no breaks, in contrast to the samples that were obtained when HAP NPs were added with a plate shape.

III-PLGA series samples (Figure 5g,h) had a porous three-dimensional structure. Figure 5g shows the internal structure of the sample III-PLGA-0.05. The sample was porous, with pore sizes ranging from 3 to 100 microns and mostly open-type pores, which can positively affect osseointegration when interacting with bone tissue. The walls of the matrix were porous, and the wall thickness on average varied from 10 to 30 microns. An increase in the concentration of HAP (Figure 5h) led to a change in the internal structure of the samples. In this case, sample III-PLGA-0.10 had elongated pores of different diameters. The structure of the sample was layered, and the wall thickness was 2–5 microns.

Studies have suggested that HAP NPs of a rod-like shape are better distributed in the resulting complex dispersed system and contribute to its aggregate stability during its hardening due to evaporation of a volatile solvent. Based on this observation, in the second series of samples (Table 1), only HAP rod-shaped nanoparticles were used.

**Figure 6:**
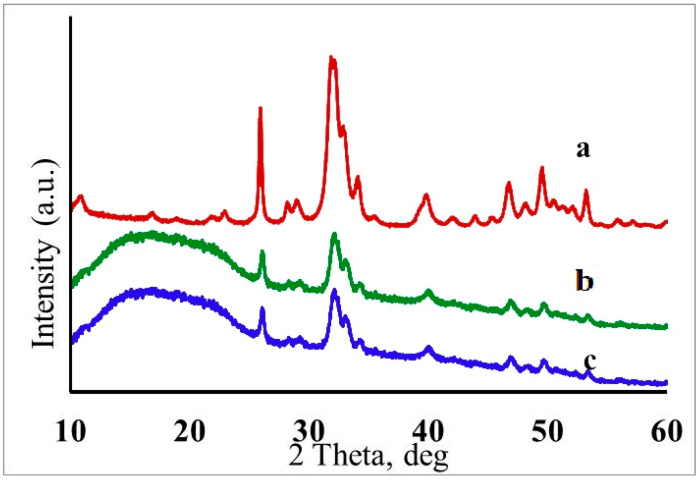
X-ray diffraction patterns of thin-film hydroxyapatite nanoparticles (**a**), III-PLGA-0.05 (**b**), and III-PLGA-0.10 (**c**).

The presence of hydroxyapatite crystals in the polymer matrix was confirmed by the results of X-ray phase analysis (Figure 6).

The X-ray diffraction pattern of the HAP powder had all characteristic peaks of the substance (PDF2 09-0432). The broadening of the peaks in the region of 30–35° 2θ angles indicated a high dispersion of the samples. The parameters of the crystal lattice were a = 9.4394 Å, c = 6.8861 Å. In this case, the parameters of elementary cells were close in their values to the reference data for stoichiometric HAP (Figure 6a). The wide halo in the diffraction patterns in the region of small angles corresponded to the polymer matrix (Figure 6b,c). The XRD results indicate that the synthesized samples contained a single crystalline phase, hydroxyapatite, which confirmed the stability of the introduced NPs.

### 3.3. Mechanical Stability and Cytotoxicity of Hydrophobic Polymer(s)/Alginate/HAP NPs Matrices

The mechanical stability and cytotoxicity of the matrices are shown in Table 2.

Nonstable samples were defined as those that completely transitioned to jelly-like consistency after 120 h; and to partially nonstable ones, those that, with a destruction area of more than 75%, retained fragments of the original matrix. A three-level identification was adopted to characterize pH: “acidic medium” at pH < 7.0; “neutral medium” at 7.0 ≤ pH ≤ 7.4 (corresponds to the manufacturer’s declaration); alkaline medium at pH > 7.4.

**Figure 7:**
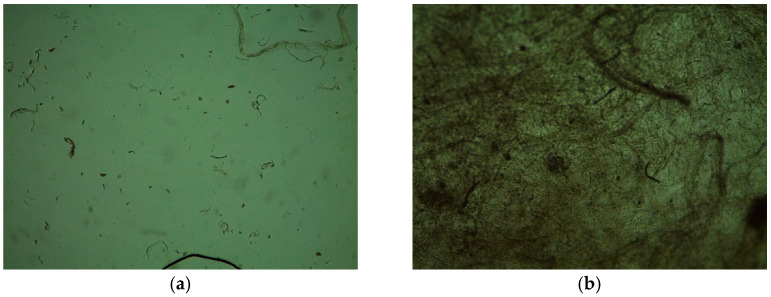
Biomaterial samples on the 5th day of cultivation (magnification × 50): (**a**)—II-PLGA-0.10, (**b**)—III-PLGA-0.10.

Significant destruction of the I-PLGA and II-PLGA samples was observed after 72 h of incubation of the matrices in phosphate-buffered saline. Samples acquired an unstable jelly-like consistency and degraded by more than 45%. Samples were destroyed by more than 50% after 120 h. Samples of the III-PLGA series were more stable, and however, even after 120 h of incubation, they were destroyed by 20–25% (Figure 7).

**Figure 8:**
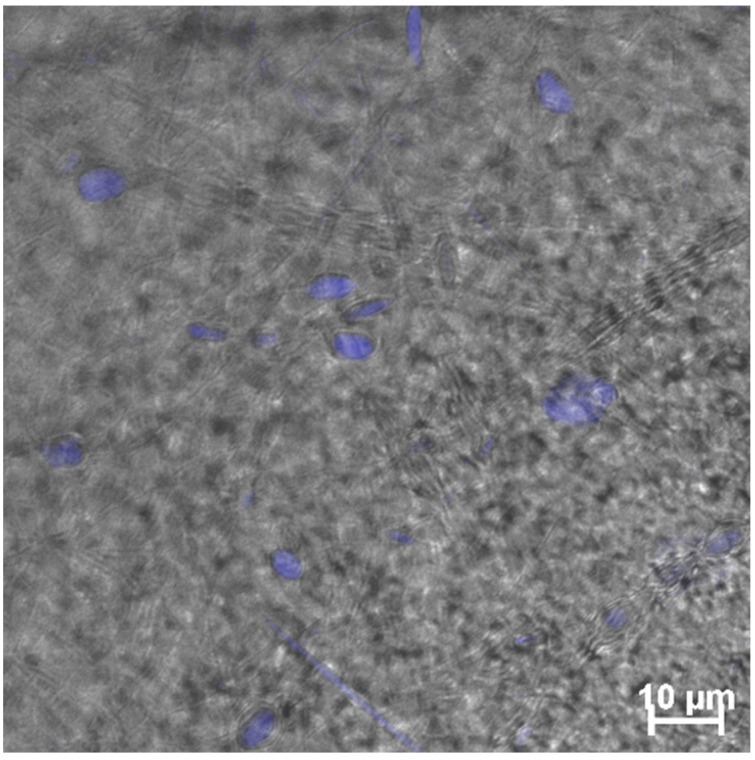
Cell culture of human fibroblasts on the surface of III-PLGA-0,10-Ca. Cultivation time is 72 h. The cell nuclei were detected by DAPI.

The cell survival in the I-PLGA and II-PLGA series varied in the range of 10–19%, and in the III-PLGA series, where mechanical stability was higher, in the range of 60–70%. Figure 8 shows the cell culture of human fibroblasts on the surface of III-PLGA-0.10-Ca. The cell nuclei were detected by DAPI.

### 3.4. Assessment of Acute Toxicity of Hydrophobic Polymer(s)/Alginate/HAP NPs Matrices

For acute toxicity studies, three samples were taken from the second part of the study, the content of hydroxyapatite nanoparticles of which was maximum (samples III-PLGA-0.10-Ca, III-PCL-0.10-Ca, and III-PLGA/PCL-0.10-Ca).

As a result of experimental mice and rats observation, there were no signs of intoxication in animals or changes in their general clinical condition compared with control mice and rats, immediately after intraperitoneal administration of the samples’ extracts III-PLGA-0.10-Ca, III-PCL-0.10-Ca, and III-PLGA/PCL-0.10-Ca, during the first 4 h, and during the subsequent 14 days observation period. During the indicated period, there was no disease or mortality of mice and rats, as well as visually detectable differences in water and feed intake in the experimental and control groups, animals were active, and behavioral reactions corresponded to indicators of species norms.

When analyzing the body mass of mice and rats during the experiment, no statistically significant differences were found between the experimental and control animals, while the dynamics of the gain was positive and corresponded to normal sex and age indices for mice and rats (Table 3).

Due to the absence of mortality of mice and rats during the entire observation period, a pathomorphological study was performed on the 15th day after the intraperitoneal administration of extracts of samples and model medium to animals.

During a pathological study of male and female mice during external examination, no differences were found between animals that received extracts of the studied samples or model environment: the coat was smooth and shiny; skin was elastic and mobile; subcutaneous tissue was moderate; visible mucous membranes were pale, clean, and without ulceration and extraneous overlays; and pathological discharge from the natural openings of the body was absent.

When opening the chest and abdominal cavities in all mice of the experimental and control groups, the anatomically correct arrangement of organs was noted, and macroscopically distinguishable signs of the pathology of the internal organs were not established. No peritoneal effusion, edema, hemorrhages, infiltrates, signs of damage to the peritoneum or organs, newly formed connective tissue, and other inflammatory and post-inflammatory changes were found at the injection specimen injection site—abdominal cavity—in the experimental mice, as well as in mice of the control group.

During a pathological study of male rats during external examination, no differences were found between animals that received extracts of the studied samples or model environment: the coat was smooth and shiny; the skin was elastic and mobile; subcutaneous tissue was moderate; visible mucous membranes were pale, clean, and without ulceration and extraneous overlays; and pathological discharge from the natural openings of the body was absent.

When opening the chest and abdominal cavities in all rats of the experimental and control rats, an anatomically correct arrangement of organs was noted, and macroscopically distinguishable signs of the pathology of the internal organs were not established. At the injection site of the extracts of samples III-PLGA-0.10-Ca, III-PCL-0.10-Ca, and III-PLGA/PCL-0.10-Ca—the abdominal cavity—no peritoneal effusion, edema, hemorrhage, infiltrates, signs of damage to the peritoneum or organs, newly formed connective tissue, and other inflammatory and post-inflammatory changes were found in the experimental rats, as well as in rats of the control group.

## 4. Discussion

The different inlet structure and mechanical stability of the samples can be explained by the influence of the shape, surface charge, and the number of added HAP nanoparticles, which act as a stabilizer in the complex dispersed system, being adsorbed at the liquid–liquid and liquid–gas interfaces.

It was shown experimentally that the shape and size of nanoparticles obtained by the method of controlled deposition in the liquid-phase HAP were significantly influenced by the concentration of surfactants. At a sodium citrate concentration of 0.05 M, the formation of agglomerates of irregularly shaped nanoplates (the average size of the plates is 30–50 nm) was observed, and with an increase in the surfactant concentration, rod-shaped nanoparticles were formed, while the average size of nanoparticles increased with the surfactant concentration. The change in the shape of nanoobjects upon the addition of a surfactant is associated with the passivation of part of the surfaces of growing crystals by surfactant molecules adsorbed on them, which slows down the growth rate of such crystal faces.

Regardless of the size and shape of the formed nanoparticles, it was experimentally shown that they were a crystalline hydroxyapatite. As the surface charge of the obtained nanoparticles was different, their ability to adsorb at the interface in a system of two immiscible phases (an aqueous solution of sodium alginate and an organic solution of PLGA) also differed. As a result, the internal structure of matrices containing HAP nanoparticles of different shapes and sizes was very different.

As shown earlier, the rod-shaped HAP nanoparticles used in the III-PLGA series provided the greater aggregate stability of the system, and it can be assumed that in this series, the distribution of polymers in the matrix volume was also more uniform. As a result, the access of the solvent to the alginate molecules was hindered by the polymeric chains of PLGA, which led to a lower rate of matrix degradation.

The transition of alginate molecules to the nutrient medium led to a shift in the pH of the nutrient medium to lower values (acidification of the medium), which in turn is one of the factors in cell death. Thus, cell survival in the I-PLGA and II-PLGA series varied in the range of 10–19%, and in the III-PLGA series, where mechanical stability was higher, in the range of 60–70%.

In the second part of the study, rod-shaped hydroxyapatite nanoparticles were used in a concentration range from 0.05 to 0.15 g per 3 mL of sodium alginate hydrogel. Additionally at this stage, in addition to PLGA, PCL (series III-PCL) or its mixture with PLGA in a mass ratio of 1:1 (series III-PLGA / PCL) was used. The expansion of the range of polymers used allowed the adjustment of the mechanical properties of the matrices and the rate of their biodegradation. In order to increase the mechanical stability of the matrices and prevent a decrease in the pH of the medium, the samples were additionally kept in an alcoholic solution of calcium chloride before freezing and drying for ionic replacement of the cations and crosslinking of polymer chains of alginate. The results of mechanical stability and cytotoxicity of the samples are shown in Table 2. The studies showed that all samples were mechanically stable during the test period and were not cytotoxic to MMSC gum cells.

The acute toxicity study of III-PLGA-0.10-Ca, III-PCL-0.10-Ca, and III-PLGA/PCL-0.10-Ca samples showed that there were no signs of acute toxicity and irritating local effects of the samples upon intraperitoneal administration of their aqueous extracts to outbred white mice and Wistar rats.

## 5. Conclusions

The porous composites consisting of a hydrophilic polymer (PLGA, PCL, or a mixture thereof), alginate, and HAP NPs as materials for tissue engineering were investigated. It was experimentally shown that the concentration of surfactants during the synthesis of hydroxyapatite nanoparticles affected the shape, size, and surface charge of the resulting HAP NPs. The type of HAP nanoparticles and their concentration significantly affected the aggregative stability of the formed dispersed “liquid–gas” system, in which the liquid was a mixture of two immiscible polymer solutions: a hydrophobic polymer in methylene chloride and hydrophilic sodium alginate in water. As a result, when the systems solidified, different porosities of the matrices were achieved. The presence of noncrosslinked alginate molecules in the matrix led to a rapid destruction of the matrices in the physiological medium, lowering the pH of the medium, which made such composites cytotoxic. Crosslinking of the alginate molecules by ionic substitution made the matrices noncytotoxic. An in vivo study showed the absence of acute toxicity of the developed composites.

## 6. Patents

Based on the results of the work, a patent RU 2019145019 “Biocompatible biodegradable scaffold based on a polymer composite containing hydroxyapatite nanoparticles” was issued.

## Figures and Tables

**Table 1:** The compositions of obtained hydrophobic polymer(s)/alginate/HAP NPs matrices.

Sample	Hydrophobic Polymer	Mass of Hydrophobic Polymer (onto 3 mL of Dichloromethane), g	Sodium Alginate Mass (onto 9 mL Water, g	Type of HAP NPs (Shape and Sizes)	Mass of HAP NPs, g
I-PLGA-0.05	PLGA	0.09	0.18	plate-shaped nanoparticles with an average thickness of 4 nm and a length of 30 to 50 nm	0.05
I-PLGA-0.10		0.09			0.10
II-PLGA-0.05		0.09		rod-shaped nanoparticles with an average diameter of 10 nm and a length of 150–180 nm	0.05
II-PLGA-0.10		0.09			0.10
III-PLGA-0.05		0.09		rod-shaped nanoparticles with an average diameter of 10 nm and a length of 110–130 nm	0.05
III-PLGA-0.10		0.09			0.10
III-PLGA-0.05-Ca		0.09			0.05
III-PLGA-0.10-Ca		0.09			0.10
III-PLGA-0.15-Ca		0.09			0.15
III-PCL-0.05-Ca	PCL	0.09			0.05
III-PCL-0.10-Ca		0.09			0.10
III-PCL-0.15-Ca		0.09			0.15
III-PLGA/PCL-0.05-Ca	PLGA	0.045			0.05
	PCL	0.045			
III-PLGA/PCL-0.10-Ca	PLGA	0.045			0.10
	PCL	0.045			
III-PLGA/PCL-0.15-Ca	PLGA	0.045			0.15
	PCL	0.045			

**Table 2:** Mechanical stability and cytotoxicity of hydrophobic polymer(s)/alginate/HAP NPs matrices.

Sample	Mechanical Stability	pH	Cytotoxicity	Cell Adhesion (12 Day)
I-PLGA-0.05	non-stable	acidic medium	cytotoxic	absent
I-PLGA-0.10	non-stable	acidic medium	cytotoxic	absent
II-PLGA-0.05	non-stable	acidic medium	cytotoxic	absent
II-PLGA-0.10	non-stable	acidic medium	cytotoxic	absent
III-PLGA-0.05	partial non-stable	neutral	low-cytotoxic	medium
III-PLGA-0.10	partial non-stable	neutral	low-cytotoxic	medium
III-PLGA-0.05-Ca	stable	neutral	non-cytotoxic	high
III-PLGA-0.10-Ca	stable	neutral	non-cytotoxic	high
III-PLGA-0.15-Ca	stable	neutral	non-cytotoxic	high
III-PCL-0.05-Ca	stable	neutral	non-cytotoxic	high
III-PCL-0.10-Ca	stable	neutral	non-cytotoxic	high
III-PCL-0.15-Ca	stable	neutral	non-cytotoxic	high
III-PLGA/PCL-0.05-Ca	stable	neutral	non-cytotoxic	high
III-PLGA/PCL-0.10-Ca	stable	neutral	non-cytotoxic	high
III-PLGA/PCL-0.15-Ca	stable	neutral	non-cytotoxic	high

**Table 3:** Body weight of experimental animals in an experiment for the study of acute toxicity.

Sample	Number of Animals	Body Weight, g		
		Initial	8 Day	14 Day
Male mouse				
Control	5	24.7 ± 1.7	29.7 ± 2.5	36.7 ± 2.2
III-PLGA-0.10-Ca	5	24.4 ± 1.9	29.3 ± 2.4	35.6 ± 2.9
III-PCL-0.10-Ca	5	24.2 ± 1.8	30.7 ± 2.4	36.9 ± 1.7
III-PLGA/PCL-0.10-Ca	5	25.0 ± 1.4	30.9 ± 1.8	35.5 ± 2.3
Female mouse				
Control	5	21.9 ± 1.2	24.8 ± 1.2	29.0 ± 1.0
III-PLGA-0.10-Ca	5	21.7 ± 1.6	24.5 ± 1.3	28.0 ± 2.4
III-PCL-0.10-Ca	5	21.5 ± 1.8	24.2 ± 1.9	28.4 ± 2.4
III-PLGA/PCL-0.10-Ca	5	21.4 ± 1.5	24.0 ± 1.8	28.1 ± 1.2
Male rats				
Control	5	204.0 ± 13.7	223.8 ± 12.9	251.4 ± 16.5
III-PLGA-0.10-Ca	5	206.2 ± 14.0	225.8 ± 10.7	255.4 ± 13.6
III-PCL-0.10-Ca	5	205.2 ± 14.1	232.6 ± 5.0	260.4 ± 15.0
III-PLGA/PCL-0.10-Ca	5	207.4 ± 14.0	229.2 ± 14.2	259.4 ± 17.8

## Data Availability

Not applicable.
